# Establishment and validation of a prediction model for small vulnerable newborns: a retrospective study

**DOI:** 10.7189/jogh.15.04337

**Published:** 2025-12-05

**Authors:** Chengqi Xiao, Chuangchuang Xu, Lijun Zhang, Dongmei Lai

**Affiliations:** 1 The International Peace Maternity and Child Health Hospital, School of Medicine, Shanghai Jiao Tong University, Shanghai, China; 2 Shanghai Key Laboratory of Embryo Original Diseases, Shanghai, China

## Abstract

**Background:**

The concept of small vulnerable newborns has been proposed, including preterm birth, low birth weight, and small for gestational age, leading causes of perinatal mortality. We aimed to identify high-risk factors for small vulnerable newborns and develop a predictive model through a retrospective analysis.

**Methods:**

We collected clinical data from pregnant women who met inclusion criteria between January 2015 and December 2023 and divided them into training and validation cohorts. We used univariate analysis and mean decreases in the Gini index to screen for potential risk factors. We applied the least absolute shrinkage and selection operator regression to select final predictors and construct a nomogram. We assessed model performance using receiver operating characteristic curves, calibration curves, and clinical decision analysis, with internal validation via 10-fold cross-validation and temporal internal validation.

**Results:**

Among 129 554 women, 13 801 (10.66%) had small vulnerable newborn, with the incidence increasing from 2015 (10.15%) to 2023 (11.61%). Key risk factors included multiple pregnancies (odds ratio (OR) = 37.2), pre-pregnancy body mass index (BMI) of <18.5 (OR = 8.61) and ≥25 kg/m^2^ (OR = 6.40), maternal age of <25 (OR = 6.81) and ≥35 years (OR = 3.72), hypertensive disorders of pregnancy (OR = 2.81), and placental disorders (OR = 3.03). Other significant factors were assisted reproductive technology, mycoplasma/chlamydia infection, and elevated bile acids. The nomogram demonstrated strong predictive performance (area under the curve = 0.873).

**Conclusions:**

The incidence of small, vulnerable newborns rose notably during 2021–2023. The developed model, incorporating age, pre-pregnancy BMI, multiple pregnancies, hypertensive disorders of pregnancy, and placental disorders, is designed to be applied in the third trimester and enables risk identification, facilitating targeted interventions to reduce neonatal mortality and complications.

**Registration:**

Chinese Clinical Trial Registry, ChiCTR2400093923

Globally, approximately 35 million babies (>25% of all births) are born too early (preterm birth (PTB)) or too small (low birth weight (LBW) or small for gestational age (SGA)) each year [1]. PTB, defined as birth before 37 weeks of gestation [2], and LBW (birth weight <2500 g) are major contributors not only to neonatal mortality [3], but also to adverse health outcomes in childhood and adolescence [4]. SGA, though variably defined (commonly using cutoffs from the 2.5th to 10th percentile), remains a key indicator for identifying high-risk newborns [5]. In 2020, an estimated 8.6 million deaths occurred from the 28th week of pregnancy up to 20 years of age, with more than half occurring within the first 28 days of life [6]. PTB, LBW, and SGA are among the leading causes of perinatal mortality [7], underscoring the urgent need for risk prediction and intervention.

Previous studies have primarily examined risk factors for PTB, LBW, and SGA as separate outcomes, revealing significant overlap in their etiologies. Common maternal risk factors include low pre-pregnancy body mass index (BMI) [8] and inadequate gestational weight gain [9]. Additionally, multiple pregnancies [10], pregnancy complications (*e.g.* hypertensive disorders of pregnancy (HDP) [11], gestational diabetes (GDM) or diabetes mellitus (DM) [12], thyroid dysfunction [13]), infections (*e.g.* syphilis [14], group B streptococcus (GBS) [15], chlamydia [16]), and placental disorders [17] further elevate the risk of PTB, LBW, and SGA.

Critically, newborns often fulfil multiple adverse criteria simultaneously (*e.g.* PTB and LBW or SGA), compounding their risk of neonatal mortality and morbidity compared to those meeting only one criterion [18]. To address this overlap, the concept of small vulnerable newborns (SVNs) has been proposed, integrating PTB, LBW, and SGA into a unified framework to improve clinical risk stratification, etiological research, and targeted interventions [7].

To investigate the associations between maternal/pregnancy-related factors and SVN, we conducted a retrospective study (2015–23) involving 129 554 pregnancies from a tertiary hospital in Southeast China. Using this data, we developed a predictive model for SVN in the third trimester, aiming to establish a comprehensive risk assessment tool for prevention.

## METHODS

### Study design and population

We conducted a single-centre observational study at the International Peace Maternity and Child Health Hospital, Shanghai Jiao Tong University School of Medicine.

We included women who were registered for delivery in the hospital between January 2015 and December 2023. We excluded those who experienced stillbirth or neonatal death, were in the <28 gestational weeks, whose newborns had chromosomal abnormalities or malformations, who had a pregnancy complicated by malignancy or history of malignancy, and those who had severe pregnancy complications (*e.g.* pulmonary hypertension, cardiac function New York Heart Association class ≥3)

### Data collection and definition

We exported all data directly from the health information case system through the hospital information department. For partially missing data, we used the random forest method for imputation to minimise the impact of data gaps. We obtained the laboratory test results from samples collected at 10–11 weeks of gestation. Data included age (<25, 25–35, ≥35 years), pre-pregnancy BMI (18.5–25, <18.5, ≥25 kg/m^2^), education (high school or below, bachelor's degree or college, postgraduate or above), blood type (A, B, O, AB), multipara (no, yes), history of multiple abortions (no, yes), assisted reproductive technology (ART) (no, yes), multiple pregnancy (no, yes), presence of *Toxoplasma gondii*, Rubella virus, Cytomegalovirus, Herpes simplex virus, (no, yes), GBS inflammation (no, yes), mycoplasma chlamydia (no, yes), viral hepatitis (no, yes), vitamin B12 deficiency (no, yes), calcium deficiency (no, yes), gestational anaemia (no, yes), gestational thrombocytopenia (no, yes), abnormal liver or kidney function (no, yes), gestational hyperuricemia (no, yes), gestational dyslipidaemia (no, yes), elevated bile acids in pregnancy (no, yes), ovarian cyst (no, yes), uterine myoma (no, yes), thyroid disorder (no, yes), HDP (no, yes), DM/GDM (no, yes), polyhydramnios (no, yes), oligohydramnios (no, yes), placental disorders (no, yes) (Table S1 in the **Online Supplementary Document**).

### SVN

SVN is a conceptualisation of a collection of newborn conditions, including PTB, SGA, and LBW. We defined PTB as birth at <37 weeks of gestation [2], SGA as birth weight <10th percentile for gestational age (foetal weight standard according to the National Institute of Child Health and Human Development, Fetal Growth Studies, Asian [19]), and LBV as birth weight <2500 g. We defined a newborn as SVN if it had any of the above pathologies.

### Statistical analysis

We chronologically divided the study population into two cohorts with an approximate 7:3 ratio: a training cohort (January 2015–December 2020) and a validation cohort (January 2021–December 2023). The initial descriptive analysis characterised the training cohort, presenting categorical variables as percentages and continuous variables as means (standard deviations).

We employed a two-stage variable selection approach. We first performed univariate analysis (*P* < 0.05 as the significance threshold) and random forest analysis to assess variable importance using mean decreased Gini (MDG) scores. We then analysed the top 50% most predictive variables from this screening using the least absolute shrinkage and selection operator (LASSO) regression to identify the most robust predictors for our final model.

For model development and validation, we constructed a nomogram based on the final LASSO regression results and performed internal 10-fold cross-validation, with performance assessed by the average area under the curve (AUC) across iterations. We also did temporal internal validation using the independent cohort, evaluated through receiver operating characteristic curve analysis (for discrimination), calibration plots (for accuracy), and decision curve analysis (for clinical utility).

We used *R*, version 4.1.1 (R Core Team, Vienna, Austria) for all analyses, with two-tailed tests and a *P* < 0.05 significance threshold.

## RESULTS

### General characteristics

During the study period, 131 727 women delivered, of whom we excluded 2173 for the following reasons: 522 cases of stillbirth or neonatal death, 71 cases with gestational age <28 weeks, 565 cases of neonatal chromosomal abnormalities or malformations, 377 cases with malignancy or history of malignancy, and 638 cases with severe pregnancy complications. This resulted in a final study population of 129 554 women who were subsequently divided into a training cohort (93 880 women) and a validation cohort (35 674 women) (Figure S1 in the **Online Supplementary Document**)

### The incidence of SVN, 2015–23

The incidence of SVN over the 2015–23 period was 10.65%, demonstrating a progressive upward trend that became particularly pronounced during 2021–23(**Table 1**; Figure S2 in the **Online Supplementary Document**). While the absolute number of SVN cases remained stable, the rising incidence rate reflects the concurrent decline in birth rates during this period. This observed increase in SVN incidence was primarily attributable to rising rates of both LBW and SGA infants.

**Table 1:** The number and incidence of SVN, 2015–23

	SVN		PTB		LBW		SGA		
**Year**	**n**	Incidence*	**n**	Incidence*	**n**	Incidence*	**n**	Incidence*	**Total n**
2015	1513	10.15	1015	6.81	690	4.63	550	3.69	14 910
2016	1711	10.20	1200	7.16	739	4.41	557	3.32	16 769
2017	1734	10.16	1142	6.69	751	4.40	647	3.79	17 062
2018	1771	11.21	1170	7.40	816	5.16	657	4.16	15 805
2019	1604	10.09	1038	6.53	728	4.58	619	3.89	15 900
2020	1378	10.26	914	6.80	596	4.44	515	3.83	13 434
2021	1401	11.58	874	7.23	639	5.28	565	4.67	12 094
2022	1264	11.18	773	6.84	605	5.35	560	4.95	11 303
2023	1425	11.61	818	6.66	676	5.51	634	5.16	12 277
Total	13 801	10.65	8944	6.90	6240	4.82	5304	4.09	129 554

LBW – low birth weight, PTB – preterm birth, SGA – small for gestational age, SVN – small vulnerable newborn

*Values presented as percentages.

### Selected factors for the model

We included 129 554 cases (13 801 SVN cases and 115 753 non-SVN cases), with missing data imputed using the random forest method. Significant risk factors for SVN included maternal age <25 (odds ratio (OR) = 6.81; 95% confidence interval (CI) = 6.36–7.29, *P* < 0.001) and ≥35 years (OR = 3.72; 95% CI = 3.58–3.86, *P* < 0.001), pre-pregnancy BMI<18.5 (OR = 8.61; 95% CI = 8.25–8.99, *P* < 0.001) and ≥25 kg/m^2^ (OR = 6.40; 95% CI = 6.10–6.72, *P* < 0.001), multiparity (OR = 1.25; 95% CI = 1.21–1.30, *P* < 0.001), history of multiple abortions (OR = 1.09; 95% CI = 1.02–1.17, *P* = 0.012), ART (OR = 2.85; 95% CI = 2.69–3.01, *P* < 0.001), multiple pregnancy (OR = 37.2; 95% CI = 34.3–40.4, *P* < 0.001), mycoplasma/chlamydia infection (OR = 2.86; 95% CI = 2.43–3.36, *P* < 0.001), vitamin B12 deficiency (OR = 1.08; 95% CI = 1.02–1.15, *P* = 0.010), abnormal liver/kidney function (OR = 1.35; 95% CI = 1.26–1.45, *P* < 0.001), gestational hyperuricemia (OR = 1.52; 95% CI = 1.21–1.89, *P* < 0.001), gestational dyslipidaemia (OR = 1.19; 95% CI = 1.14–1.24, *P* < 0.001), elevated bile acids (OR = 2.85; 95% CI = 2.54–3.18, *P* < 0.001), uterine myoma (OR = 1.07; 95% CI = 1.00–1.14, *P* = 0.048), thyroid disorders (OR = 1.07; 95% CI = 1.01–1.14, *P* = 0.024), HDP (OR = 2.81; 95% CI = 2.67–2.96, *P* < 0.001), DM/GDM (OR = 1.26; 95% CI = 1.20–1.31, *P* < 0.001), oligohydramnios (OR = 1.83; 95% CI = 1.66–2.01, *P* < 0.001), and placental disorders (OR = 3.03; 95% CI = 2.83–3.24, *P* < 0.001) (**Table 2**). Significant protective factors for SVN were education level of junior college/university (OR = 0.80; 95% CI = 0.76–0.86, *P* < 0.001) and graduate degree (OR = 0.71; 95% CI = 0.66–0.76, *P* < 0.001), GBS inflammation (OR = 0.81; 95% CI = 0.75–0.88, *P* < 0.001), calcium deficiency (OR = 0.75; 95% CI = 0.63–0.89, *P* = 0.002), gestational anaemia (OR = 0.93; 95% CI = 0.88–0.97, *P* = 0.002).

**Table 2:** Characteristics of the SVN at baseline*

	All (n = 129 554)	No SVN (n = 115 753)	SVN (n = 13 801)	OR (95% CI)	*P-*value†
**Age**					<0.001
25–35	94 724 (73.1)	88 576 (76.5)	6148 (44.5)	ref	
<25	4365 (3.37)	2964 (2.56)	1401 (10.2)	6.81 (6.36–7.29)	
≥35	30 465 (23.5)	24 213 (20.9)	6252 (45.3)	3.72 (3.58–3.86)	
**Pre-pregnancy BMI**					<0.001
18.5–25	95 685 (73.9)	91 175 (78.8)	4510 (32.7)	ref	
<18.5	19 644 (15.2)	13 775 (11.9)	5869 (42.5)	8.61 (8.25–8.99)	
≥25	14 225 (11.0)	10 803 (9.33)	3422 (24.8)	6.40 (6.10–6.72)	
Missing	10 328	8800	1528		
**Education**					<0.001
High school or below	9696 (7.48)	8437 (7.29)	1259 (9.12)	ref	
Junior college or university	94 025 (72.6)	83 951 (72.5)	10 074(73.0)	0.80 (0.76–0.86)	
Graduate or above	25 833 (19.9)	23 365 (20.2)	2468 (17.9)	0.71 (0.66–0.76)	
Missing	10 300	8786	1514		
**Blood type**					0.256
A	46 964 (36.3)	41 905 (36.2)	5059 (36.7)	ref	
B	36 346 (28.1)	32 424 (28.0)	3922 (28.4)	1.00 (0.96–1.05)	
O	5191 (4.01)	4645 (4.01)	546 (3.96)	0.97 (0.89–1.07)	
AB	41 053 (31.7)	36 779 (31.8)	4274 (31.0)	0.96 (0.92–1.00)	
Missing	1616	1457	159		
**Multipara**					<0.001
No	89 955 (69.4)	80 984 (70.0)	8971 (65.0)	ref	
Yes	39 599 (30.6)	34 769 (30.0)	4830 (35.0)	1.25 (1.21–1.30)	
**History of multiple abortions**					0.012
No	120 537 (93.0)	107 768 (93.1)	12 769 (92.5)	ref	
Yes	9017 (6.96)	7985 (6.90)	1032 (7.48)	1.09 (1.02–1.17)	
**ART**					<0.001
No	121 921 (94.1)	109 929 (95.0)	11 992 (86.9)	ref	
Yes	7633 (5.89)	5824 (5.03)	1809 (13.1)	2.85 (2.69–3.01)	
**Multiple pregnancy**					<0.001
No	126 112 (97.3)	115 004 (99.4)	11 108 (80.5)	ref	
Yes	3442 (2.66)	749 (0.65)	2693 (19.5)	37.2 (34.3–40.4)	
**TORCH**					0.657
No	129 189 (99.7)	115 430 (99.7)	13 759 (99.7)	ref	
Yes	365 (0.28)	323 (0.28)	42 (0.30)	1.09 (0.78–1.49)	
Missing	5972	5119	853		
**GBS inflammation**					<0.001
No	121 825 (94.0)	108 711 (93.9)	13 114 (95.0)	ref	
Yes	7729 (5.97)	7042 (6.08)	687 (4.98)	0.81 (0.75–0.88)	
**Mycoplasma chlamydia**					<0.001
No	128 778 (99.4)	115 173 (99.5)	13 605 (98.6)	ref	
Yes	776 (0.60)	580 (0.50)	196 (1.42)	2.86 (2.43–3.36)	
**Viral hepatitis**					0.064
No	126 240 (97.4)	112 759 (97.4)	13 481 (97.7)	ref	
Yes	3314 (2.56)	2994 (2.59)	320 (2.32)	0.89 (0.79–1.00)	
**Vitamin B12 deficiency**					0.010
No	117 889 (91.0)	105 413 (91.1)	12 476 (90.4)	ref	
Yes	11 665 (9.00)	10 340 (8.93)	1325 (9.60)	1.08 (1.02–1.15)	
Missing	20 901	18 512	2389		
**Calcium deficiency**					0.002
No	127 873 (98.7)	114 211 (98.7)	13 662 (99.0)	ref	
Yes	1681 (1.30)	1542 (1.33)	139 (1.01)	0.75 (0.63–0.89)	
Missing	7913	6569	1344		
**Gestational anaemia**					0.002
No	107 419 (82.9)	95 847 (82.8)	11 572 (83.8)	ref	
Yes	22 135 (17.1)	19 906 (17.2)	2229 (16.2)	0.93 (0.88–0.97)	
Missing	4070	3457	613		
**Gestational thrombocytopenia**					0.369
No	129 322 (99.8)	115 541 (99.8)	13 781 (99.9)	ref	
Yes	232 (0.18)	212 (0.18)	20 (0.14)	0.80 (0.49–1.23)	
Missing	6089	5098	1191		
**Abnormal liver or kidney function**					<0.001
No	123 057 (95.0)	110 145 (95.2)	12 912 (93.6)	ref	
Yes	6497 (5.01)	5608 (4.84)	889 (6.44)	1.35 (1.26–1.45)	
Missing	7217	5944	1273		
**Gestational hyperuricemia**					<0.001
No	128 946 (99.5)	115 238 (99.6)	13 708 (99.3)	ref	
Yes	608 (0.47)	515 (0.44)	93 (0.67)	1.52 (1.21–1.89)	
Missing	7344	6053	1291		
**Gestational dyslipidaemia**					<0.001
No	93 756 (72.4)	84 207 (72.7)	9549 (69.2)	ref	
Yes	35 798 (27.6)	31 546 (27.3)	4252 (30.8)	1.19 (1.14–1.24)	
Missing	7859	6520	1339		
**Elevated bile acids in pregnancy**					<0.001
No	127 891 (98.7)	114 505 (98.9)	13 386 (97.0)	ref	
Yes	1663 (1.28)	1248 (1.08)	415 (3.01)	2.85 (2.54–3.18)	
Missing	331	240	91		
**Ovarian cyst**					0.883
No	126 136 (97.4)	112 696 (97.4)	13 440 (97.4)	ref	
Yes	3418 (2.64)	3057 (2.64)	361 (2.62)	0.99 (0.89–1.10)	
**Uterine myoma**					0.048
No	119 402(92.2)	106 742(92.2)	12 660(91.7)	ref	
Yes	10 152 (7.84)	9011 (7.78)	1141 (8.27)	1.07 (1.00–1.14)	
**Thyroid disorder**					0.024
No	117 211 (90.5)	104 799 (90.5)	12 412 (89.9)	ref	
Yes	12 343 (9.53)	10 954 (9.46)	1389 (10.1)	1.07 (1.01–1.14)	
**HDP**					<0.001
No	119 396 (92.2)	107 931 (93.2)	11 465 (83.1)	ref	
Yes	10 158 (7.84)	7822 (6.76)	2336 (16.9)	2.81 (2.67–2.96)	
**DM/GDM**					<0.001
No	108 342 (83.6)	97 208 (84.0)	11 134 (80.7)	ref	
Yes	21 212 (16.4)	18 545 (16.0)	2667 (19.3)	1.26 (1.20–1.31)	
**Polyhydramnios**					0.052
No	128 536 (99.2)	114 863 (99.2)	13 673 (99.1)	ref	
Yes	1018 (0.79)	890 (0.77)	128 (0.93)	1.21 (1.00–1.45)	
**Oligohydramnios**					<0.001
No	126 704 (97.8)	113 406 (98.0)	13 298 (96.4)	ref	
Yes	2850 (2.20)	2347 (2.03)	503 (3.64)	1.83 (1.66–2.01)	
**Placental disorders**					<0.001
No	124 780 (96.3)	112 190 (96.9)	12 590 (91.2)	ref	
Yes	4774 (3.68)	3563 (3.08)	1211 (8.77)	3.03 (2.83–3.24)	

ART – assisted reproductive technology, BMI – body mass index, CI – confidence interval, DM – diabetes mellitus, GBS – group B streptococcus, GDM – gestational diabetes mellitus, HDP – hypertensive disorders of pregnancy, OR – odds ratio, ref – reference, SVN – small vulnerable newborn, TORCH – *Toxoplasma gondii*, other infections, rubella, cytomegalovirus, herpes simplex virus

*Values are presented as n (%) unless specified otherwise.

†*P*-values are from a χ^2^ test.

We then divided the study population into a training cohort (n = 93 880) comprising women who delivered between January 2015 and December 2020, and a validation cohort (n = 35 674) consisting of deliveries from January 2021 to December 2023 (Table S2 in the **Online Supplementary Document**). Using the random forest algorithm with 100 trees, we calculated MDG values to assess variable importance in the training cohort. The top 50% of factors by MDG ranking included multiple gestation (MDG = 1842.64), pre-pregnancy BMI (MDG = 1782.22), age (MDG = 1005.46), blood type (MDG = 252.06), multiparity (MDG = 241.85), education (MDG = 213.82), ART (MDG = 188.69), placental disorders (MDG = 169.85), HDP (MDG = 164.93), gestational dyslipidaemia (MDG = 120.25), gestational anaemia (MDG = 105.27), DM/GDM (MDG = 102.46), uterine myoma (MDG = 91.88), and thyroid disorders (MDG = 89.82) (Figure S3 in the **Online Supplementary Document**). Based on both the top 50% MDG ranking and statistical significance (*P* < 0.05), we ultimately identified 12 key factors significantly associated with SVN: multiple pregnancy, pre-pregnancy BMI, age, multiparity, education level, ART use, placental disorders, HDP, gestational dyslipidaemia, gestational anaemia, DM/GDM, uterine myoma, and thyroid disorders.

Our LASSO regression analysis selected the optimal lambda value at one standard error above the minimum mean squared error, identifying five key variables that were subsequently confirmed in multivariate analysis to be strongly associated with SVN occurrence (Figure S4 in the **Online Supplementary Document**). These included maternal age <25 years (adjusted OR (aOR) = 5.60; 95% CI = 5.09–6.16, *P* < 0.001) and ≥35 years (aOR = 4.07; 95% CI = 3.86–4.30, *P* < 0.001) (compared to 25–35-year reference), pre-pregnancy BMI<18.5 (aOR = 9.73; 95% CI = 9.18–10.31, *P* < 0.001) and ≥25 kg/m^2^ (aOR = 5.41; 95% CI = 5.06–5.78, *P* < 0.001) (compared to 18.5–25 kg/m^2^ reference); multiple gestation (aOR = 35.48; 95% CI = 31.84–39.60, *P* < 0.001), HDP (aOR = 2.02; 95% CI = 1.86–2.18, *P* < 0.001), and placental disorders (aOR = 3.32; 95% CI = 2.99–3.68, *P* < 0.001) (**Table 3**). All associations demonstrated highly significant statistical relationships with SVN risk.

**Table 3:** Variables associated with SVN in multivariate analyses

	aOR (95% CI)	*P-*value*
**Age**		
25–35	ref	ref
<25	5.60 (5.09–6.16)	<0.001
≥35	4.07 (3.86–4.30)	<0.001
**Pre-pregnancy BMI**		
18.5–25	ref	ref
<18.5	9.73 (9.18–10.31)	<0.001
≥25	5.41 (5.06–5.78)	<0.001
**Multiple pregnancy**		
No	ref	ref
Yes	35.48 (31.84–39.60)	<0.001
**HDP**		
No	ref	ref
Yes	2.02 (1.86–2.18)	<0.001
**Placental disorders**		
No	ref	ref
Yes	3.32 (2.99–3.68)	<0.001

aOR – adjusted odds ratio, BMI – body mass index, CI – confidence interval, HDP – hypertensive disorders of pregnancy, ref – reference, SVN – small vulnerable newborn

*P-values are from a logistic regression analysis.

### Risk prediction nomogram establishment

**Figure 1:**
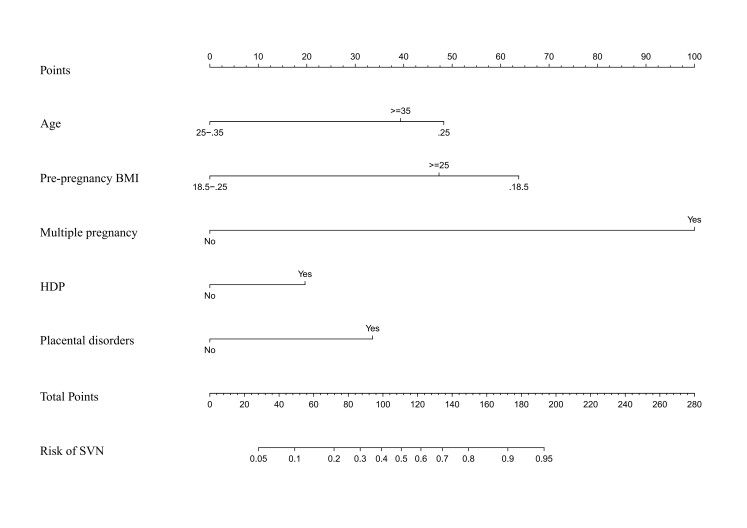
SVN risk nomogram legend. Each predictor is assigned a score on each axis. The sum of all points for all predictors is computed and denoted as the total score. BMI – body mass index, HDP – hypertensive disorders of pregnancy, SVN – small vulnerable newborn.

In the final predictive model, we incorporated five key variables (age, pre-pregnancy BMI, multiple pregnancy, HDP, and placental disorders) to construct a clinically practical nomogram for SVN risk assessment. Each risk factor was assigned weighted points on a standardised scale based on its regression coefficient, with higher-risk categories (such as younger age, extreme BMI values, or presence of complications) receiving proportionally greater point values (**Figure 1**).

### Performance of the nomogram

**Figure 2:**
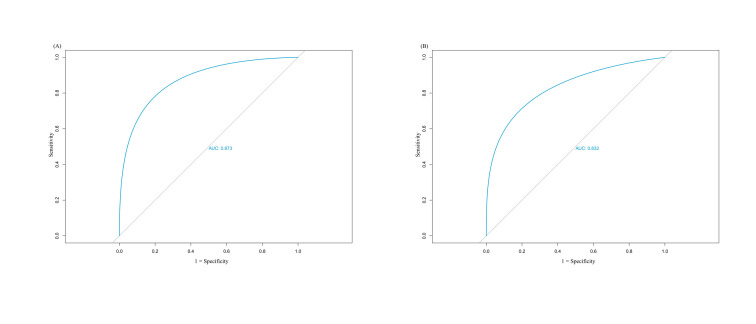
ROC for the prediction model. **Panel A.** Training cohort. **Panel B.** Validation cohort. AUC – area under curve, ROC – receiver operating characteristic curve.

We assessed the nomogram's discriminatory power for SVN prediction using receiver operating characteristic curve analysis. The AUCs were 0.873 (95% CI = 0.845–0.884) in the training cohort and 0.832 (95% CI = 0.821–0.841) in the validation cohort (**Figure 2****,** Panels A and B). Calibration analysis revealed good agreement between predicted and observed probabilities in both cohorts, with calibration curves closely following the ideal line (Figure S5 in the **Online Supplementary Document**). Decision curve analysis indicated clinical utility across threshold probabilities ranging from 10% to 90% in both cohorts (Figure S6 in the **Online Supplementary Document**). Internal validation through 10-fold cross-validation yielded an accuracy of 91.3% and a Kappa coefficient of 0.353. These results indicate that the model demonstrates satisfactory discriminative performance for SVN prediction.

## DISCUSSION

We observed an increasing incidence of SVN during 2015–23, with a notable rise from 2021 to 2023 in a maternity hospital in Shanghai. The observed increase in SVN incidence appears primarily attributable to rising rates of SGA and LBW infants. Multiple factors showed significant associations with SVN risk, including maternal characteristics (age, pre-pregnancy BMI, education level, and parity), reproductive history (multiple abortions and ART use), pregnancy complications (multiple gestation, GBS inflammation, and mycoplasma/chlamydia infection), nutritional status (vitamin B12 deficiency, calcium deficiency, and gestational anaemia), metabolic factors (abnormal liver/kidney function, hyperuricemia, dyslipidaemia, and elevated bile acids), and obstetric conditions (uterine myoma, thyroid disorders, HDP, DM/GDM, oligohydramnios, and placental disorders). We observed lower-than-expected odds ratios for GBS infection (OR = 0.81), calcium deficiency (OR = 0.75), and anaemia (OR = 0.93). Specific confounding factors may influence these findings. For GBS, the data may be incomplete because standard hospital practice is to collect samples at 34–36 weeks of gestation, and some SVN records lack this information. For calcium and iron deficiency, heightened patient and provider awareness from early pregnancy may lead to widespread supplementation, artificially reducing the observed prevalence of these deficiencies later.

Using feature selection based on MDG values and univariate analysis results, we identified five key predictors for inclusion in the model: maternal age, pre-pregnancy BMI, multiple pregnancy, HDP, and placental disorders. The developed nomogram aimed at primary care for all pregnancies demonstrated robust predictive capability, achieving an AUC of 0.873 (95% CI = 0.845–0.884) in the training cohort. Model validation showed satisfactory calibration and clinical utility across probability thresholds from 10% to 90%. Temporal internal validation in an independent cohort yielded an AUC of 0.832 (95% CI = 0.821–0.841). Internal cross-validation results (accuracy = 91.3%; Kappa = 0.353; sensitivity = 0.992; specificity = 0.719; precision = 0.916; F1 score = 0.953) further confirmed the model's discriminative performance. These findings suggest the nomogram may serve as a clinically useful tool for SVN risk assessment.

Epidemiological studies demonstrate a U-shaped relationship between maternal age and PTB risk, with the lowest PTB rates occurring among women aged 25–29 years for singleton pregnancies and 30–34 years for multiple pregnancies [20]. Our findings corroborate this pattern, showing both younger (<25 years) and advanced maternal age (≥35 years) as significant predictive factors for SVN. Maternal nutritional status similarly shows a U-shaped risk association, with pre-pregnancy BMI of <18.5 kg/m^2^ conferring elevated PTB risk independent of gestational weight gain, race, or ethnicity, the BMI of 35.0–39.9 kg/m^2^ increases PTB risk by 93% (OR = 1.93; 95%CI = 1.62–2.30) [21]. Multiple gestation represents another major risk axis, with PTB rates escalating significantly from singletons to twins to higher-order multiples, accompanied by progressively higher incidence of SGA and LBW infants [22]. Hypertensive disorders of pregnancy, particularly persistent hypertension, demonstrate strong associations with adverse outcomes [23], as do various placental pathologies, including placenta previa [24] and succenturiate placenta [25]. Notably, most identified SVN risk factors represent established, independent predictors of PTB, SGA, and LBW. This substantial overlap suggests the SVN construct may serve as a clinically valuable umbrella category, enabling unified management approaches for newborns sharing these common pathogenic pathways while facilitating targeted prevention strategies. It is crucial to emphasise that our model is strictly predictive and does not imply causation. While the identified features are strong statistical predictors of the outcome, they should not be misinterpreted as direct causes.

A systematic review of adverse pregnancy outcome prediction models highlights significant limitations, including geographical imbalance (only nine studies from low- and middle-income countries), inadequate external validation (merely ten models externally validated), and variable performance across outcomes – with LBW models (AUC = 0.60–0.84) outperforming PTB (AUC = 0.51–0.83) and SGA (AUC = 0.54–0.81) predictions [26]. Addressing these gaps, we leverage a large, diverse sample (n = 129 554) and integrate multidimensional predictors – demographic, clinical, and laboratory data – to develop a robust SVN prediction model. This approach not only enhances generalisability but also represents the first tailored tool for SVN identification and referral in Southeast China's primary healthcare system.

Our model leverages a large data set and advances the field by combining comprehensive risk factor assessment with validation (training cohort AUC = 0.873; validation cohort AUC = 0.832). By incorporating population characteristics, pregnancy complications, and laboratory metrics, we provide a practical framework for risk stratification in resource-limited settings. The findings are particularly relevant for Chinese healthcare, where timely identification of high-risk pregnancies can optimise resource allocation and neonatal outcomes. This model can assist in screening within primary care to facilitate referral to higher-level medical units.

Despite these strengths, our single-centre (Shanghai), retrospective design may limit generalisability to rural or socioeconomically diverse regions of China. Therefore, our study lacks external validation. While we mitigated bias through strict quality control, residual confounding and broad disease categorisations persist. Unexplored risk domains – detrimental addiction (smoking, alcohol use, substance abuse [27–29]), environmental exposures (noise, stress, radiation [30–32]), genetic/epigenetic factors, and microbiome interactions – warrant investigation in prospective, multicentre studies to refine SVN prediction and elucidate underlying mechanisms. The retrospective nature of the data limits precise ascertainment of the timing (gestational week) of certain critical factors, thereby precluding the construction of a time-series prediction model anchored to specific gestational milestones. Future research should prioritise these avenues to strengthen clinical applicability across populations.

## CONCLUSIONS

We found a significant increasing trend in SVN incidence during 2015–23. We have successfully developed and validated a predictive model incorporating five key clinical factors: maternal age, pre-pregnancy BMI, multiple pregnancy, HDP, and placental disorders. This model serves as an effective tool for identifying SVN risk in the third trimester, facilitating timely clinical intervention and referral. By enabling targeted preventive measures, our findings contribute to improved pregnancy outcomes and neonatal health management.

## Additional material


Online Supplementary Document
