# Supplementary material for: Establishment and validation of a prediction model for small vulnerable newborns: a retrospective study
Source: J Glob Health. 2025 Dec 5;15:04337. doi: 10.7189/jogh.15.04337 (PMC12677243; doi:10.7189/jogh.15.04337)
Supplement: Online Supplementary Document [file jogh-15-04337-s001.pdf]

Supplement to: Xiao C, Xu C, Zhang L, Lai D. Establishment and validation of a prediction model for small vulnerable newborns: a retrospective study. J Glob Health. 2025;15:04337.

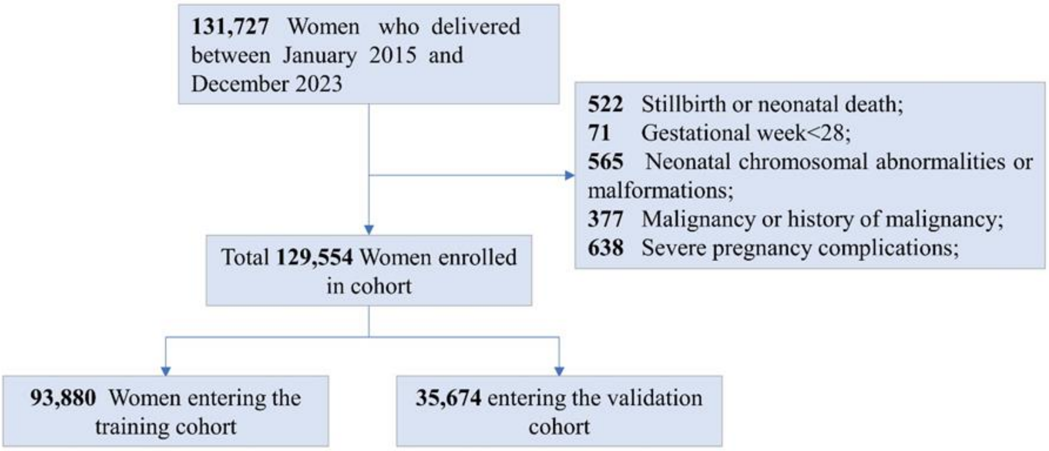

eFig 1. Flow chart for identification of eligible study population.

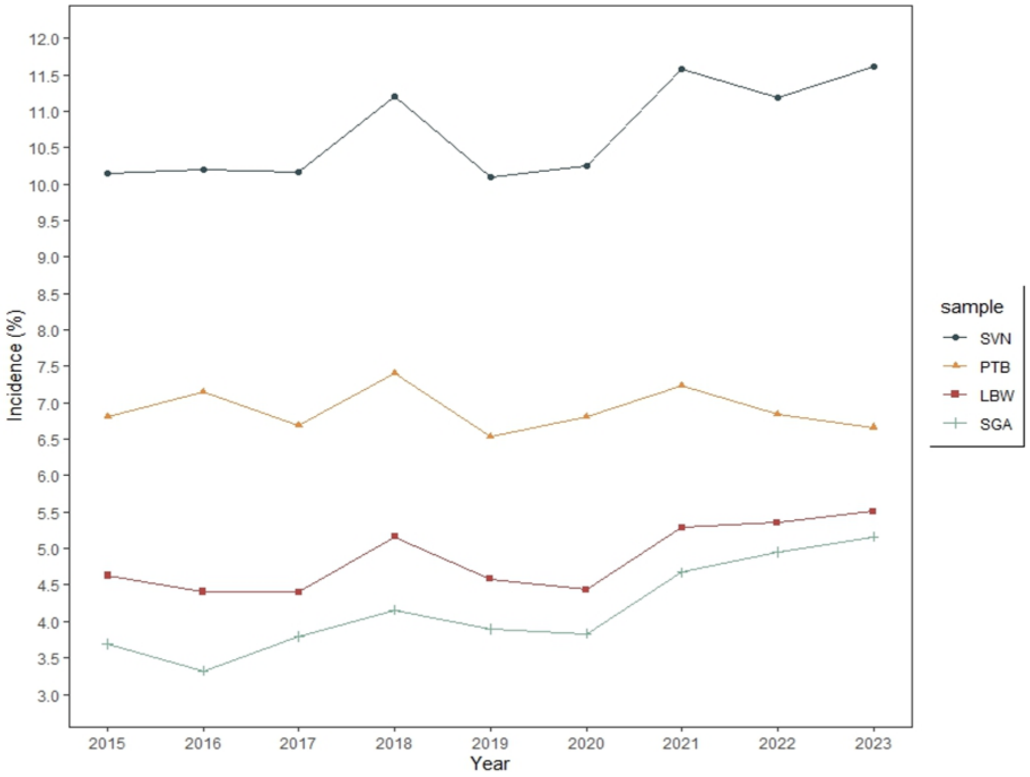

SVN: Small vulnerable newborn; PTB: Preterm birth; SGA: Small for gestational age; LBW: Low birth weight

eFig 2. The incidence of SVN from 2015 to 2023.

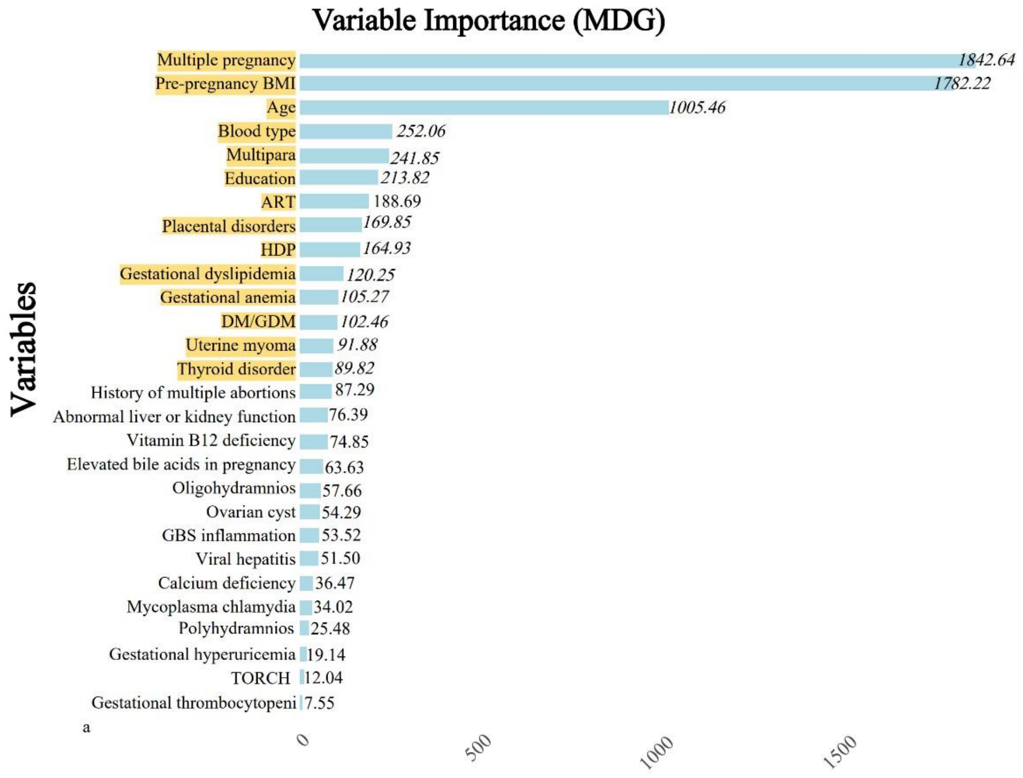

eFig 3. MDG values of each variable

BMI: Body mass index; ART: Assisted reproductive technology; GBS: Group B Streptococcus; HDP: Hypertensive disorders of pregnancy; DM: Diabetes mellitus; GDM: Gestational diabetes mellitus.

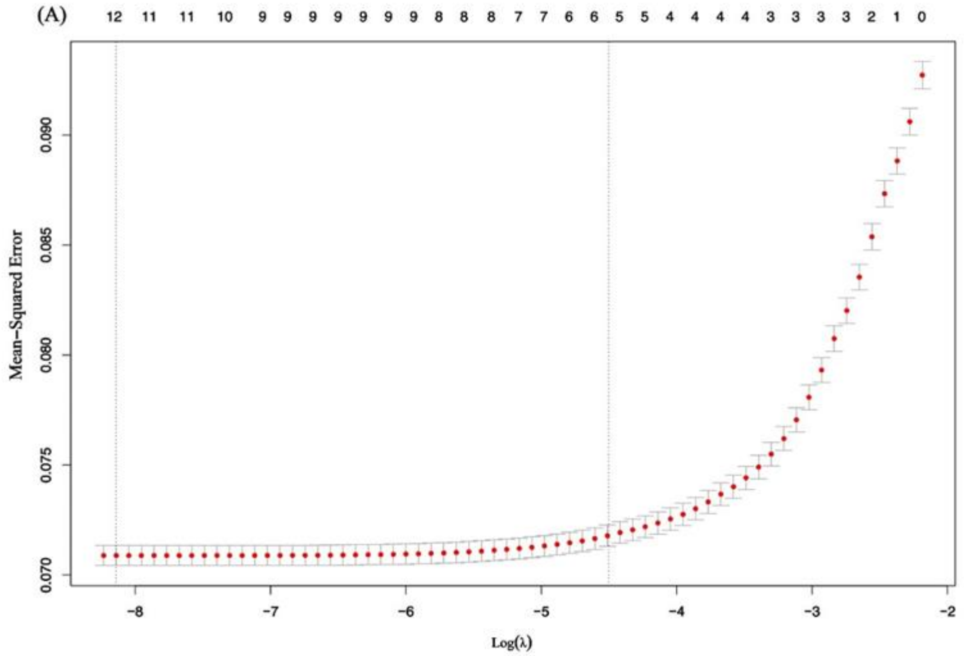

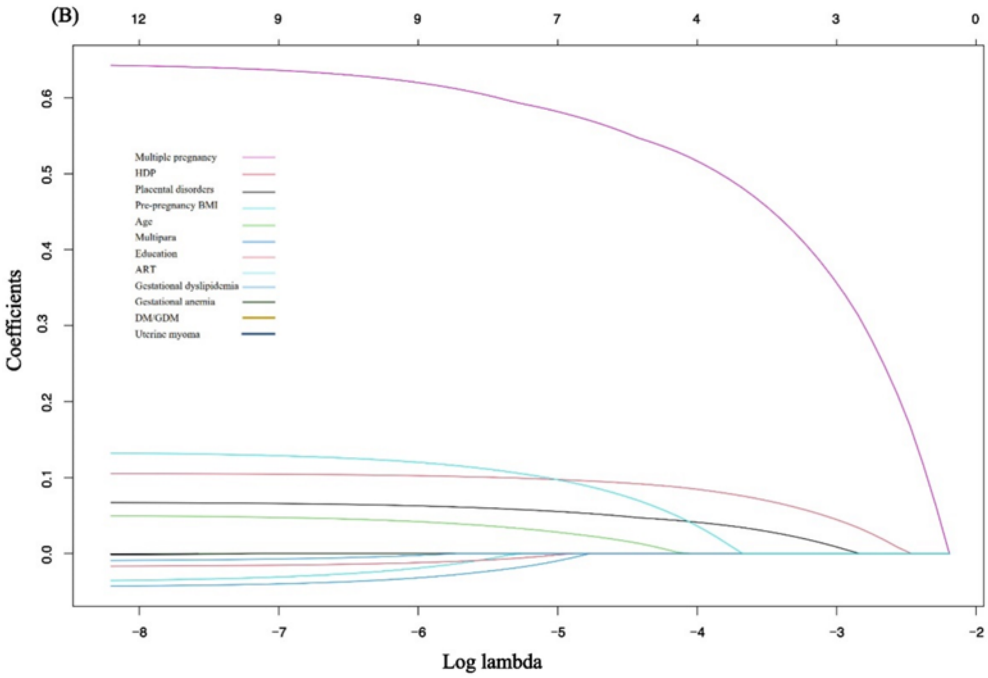

eFig 4. LASSO regression analysis a. Cross-validation curve; b. coefficient distribution plot;

BMI: Body mass index; ART: Assisted reproductive technology; GBS: Group B Streptococcus; HDP: Hypertensive disorders of pregnancy; DM: Diabetes mellitus; GDM: Gestational diabetes mellitus.

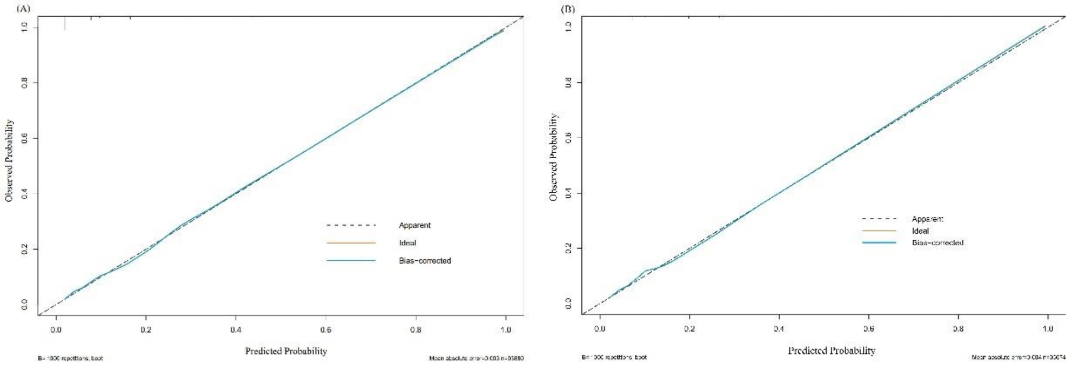

eFig 5. Calibration plot of the mode; a. Training cohort; b. Validation cohort

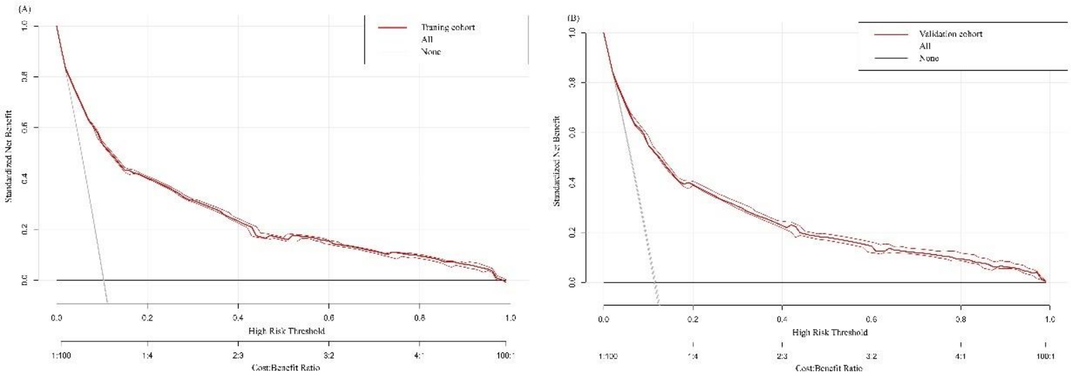

eFig 6 Decision curve analysis for SVN. a. Training cohort; b. Validation cohort;

The decision curve analysis shows that if the threshold is between 0.1–0.9, use of the nomogram in this study to predict small vulnerable newborn adds more benefit.

eTable1 Variables Categorization

Variables Categorization	Variables
Demographic and Socioeconomic Characteristics	Age<br>Pre-BMI:<br>Education<br>Blood type
Obstetric and Reproductive History	Multipara<br>History of multiple abortions<br>IVF
Current Pregnancy Characteristics and Complications	Multiple pregnancy<br>TORCH<br>GBS inflammation<br>Mycoplasma chlamydia<br>Viral hepatitis<br>Vitamin B12 deficiency<br>Calcium deficiency<br>Gestational anemia<br>Gestational thrombocytopenia<br>Abnormal liver or kidney function<br>Gestational hyperuricemia<br>Gestational dyslipidemia<br>Elevated bile acids in pregnancy<br>Ovarian cyst<br>Uterine myoma<br>Thyroid disorder<br>HDP<br>DM/GDM<br>Polyhydramnios<br>Oligohydramnios<br>Placental disorders

BMI: Body mass index; ART: Assisted reproductive technology; GBS: Group B Streptococcus; HDP: Hypertensive disorders of pregnancy; DM: Diabetes mellitus; GDM: Gestational diabetes mellitus

eTable2 Characteristics of the Training and Validation Cohorts

	Training Cohorts				Validation Cohorts			
	All	SVN		*p*	All	SVN		*p*
	N=93880	No N=84169	yes N=9711		N=35674	No N=31584	yes N=4090	
**Age (%)**				<0.001[a]				<0.001[a]
25-<35	69649 (74.2)	65275 (77.6)	4374 (45.0)		25075 (70.3)	23301 (73.8)	1774 (43.4)	
<25	3373 (3.59)	2361 (2.81)	1012 (10.4)		992 (2.78)	603 (1.91)	389 (9.51)	
⩾35	20858 (22.2)	16533 (19.6)	4325 (44.5)		9607 (26.9)	7680 (24.3)	1927 (47.1)	
**Pre-pregnancy BMI (%)**				<0.001[a]				<0.001[a]
18.5-<25	69480 (74.0)	66331 (78.8)	3149 (32.4)		26205 (73.5)	24844 (78.7)	1361 (33.3)	
<18.5	14592 (15.5)	10398 (12.4)	4194 (43.2)		5052 (14.2)	3377 (10.7)	1675 (41.0)	
⩾25	9808 (10.4)	7440 (8.84)	2368 (24.4)		4417 (12.4)	3363 (10.6)	1054 (25.8)	
**Education (%)**				<0.001[a]				<0.001[a]
High school or low	7108 (7.57)	6203 (7.37)	905 (9.32)		2588 (7.25)	2234 (7.07)	354 (8.66)	
Junior college or university	68960 (73.5)	61785 (73.4)	7175 (73.9)		25065 (70.3)	22166 (70.2)	2899 (70.9)	
Graduate or above	17812 (19.0)	16181 (19.2)	1631 (16.8)		8021 (22.5)	7184 (22.7)	837 (20.5)	
**Blood type (%)**				0.341[a]				0.213[a]
A	32780 (34.9)	29358 (34.9)	3422 (35.2)		14184 (39.8)	12547 (39.7)	1637 (40.0)	

B	26217 (27.9)	23472 (27.9)	2745 (28.3)		10129 (28.4)	8952 (28.3)	1177 (28.8)	
O	5119 (5.45)	4576 (5.44)	543 (5.59)		72 (0.20)	69 (0.22)	3 (0.07)	
AB	29764 (31.7)	26763 (31.8)	3001 (30.9)		11289 (31.6)	10016 (31.7)	1273 (31.1)	
**Multipara (%)**				<0.001[a]				<0.001[a]
No	64771 (69.0)	58921 (70.0)	5850 (60.2)		25184 (70.6)	22063 (69.9)	3121 (76.3)	
Yes	29109 (31.0)	25248 (30.0)	3861 (39.8)		10490 (29.4)	9521 (30.1)	969 (23.7)	
**History of multiple abortions (%)**				0.334[a]				0.004[a]
No	87549 (93.3)	78516 (93.3)	9033 (93.0)		32988 (92.5)	29252 (92.6)	3736 (91.3)	
Yes	6331 (6.74)	5653 (6.72)	678 (6.98)		2686 (7.53)	2332 (7.38)	354 (8.66)	
**ART (%)**				<0.001[a]				<0.001[a]
No	88355 (94.1)	80026 (95.1)	8329 (85.8)		33566 (94.1)	29903 (94.7)	3663 (89.6)	
Yes	5525 (5.89)	4143 (4.92)	1382 (14.2)		2108 (5.91)	1681 (5.32)	427 (10.4)	
**Multiple pregnancy (%)**				<0.001[a]				<0.001[a]
No	91268 (97.2)	83563 (99.3)	7705 (79.3)		34844 (97.7)	31441 (99.5)	3403 (83.2)	
Yes	2612 (2.78)	606 (0.72)	2006 (20.7)		830 (2.33)	143 (0.45)	687 (16.8)	
**TORCH (%)**				0.667 [a]				0.180[a]
No	93643 (99.7)	83954 (99.7)	9689 (99.8)		35546 (99.6)	31476 (99.7)	4070 (99.5)	
Yes	237 (0.25)	215 (0.26)	22 (0.23)		128 (0.36)	108 (0.34)	20 (0.49)	
**GBS inflammation (%)**				<0.001[a]				0.128[a]

No	88961 (94.8)	79640 (94.6)	9321 (96.0)		32864 (92.1)	29071 (92.0)	3793 (92.7)	
Yes	4919 (5.24)	4529 (5.38)	390 (4.02)		2810 (7.88)	2513 (7.96)	297 (7.26)	
**Mycoplasma chlamydia (%)**				<0.001[a]				<0.001[a]
No	93526 (99.6)	83908 (99.7)	9618 (99.0)		35252 (98.8)	31265 (99.0)	3987 (97.5)	
Yes	354 (0.38)	261 (0.31)	93 (0.96)		422 (1.18)	319 (1.01)	103 (2.52)	
**Viral hepatitis (%)**				0.588[a]				0.008[a]
No	91095 (97.0)	81663 (97.0)	9432 (97.1)		35145 (98.5)	31096 (98.5)	4049 (99.0)	
Yes	2785 (2.97)	2506 (2.98)	279 (2.87)		529 (1.48)	488 (1.55)	41 (1.00)	
**Vitamin B12 deficiency (%)**				0.020[a]				0.733[a]
No	89356 (95.2)	80160 (95.2)	9196 (94.7)		28533 (80.0)	25253 (80.0)	3280 (80.2)	
Yes	4524 (4.82)	4009 (4.76)	515 (5.30)		7141 (20.0)	6331 (20.0)	810 (19.8)	
**Calcium deficiency (%)**				0.008[a]				0.248[a]
No	92216 (98.2)	82644 (98.2)	9572 (98.6)		35657 (100.0)	31567 (99.9)	4090 (100)	
Yes	1664 (1.77)	1525 (1.81)	139 (1.43)		17 (0.05)	17 (0.05)	0 (0.00)	
**Gestational anemia (%)**				0.004[a]				0.227[a]
No	77708 (82.8)	69570 (82.7)	8138 (83.8)		29711 (83.3)	26277 (83.2)	3434 (84.0)	
Yes	16172 (17.2)	14599 (17.3)	1573 (16.2)		5963 (16.7)	5307 (16.8)	656 (16.0)	
**Gestational thrombocytopenia (%)**				0.549[a]				0.605[a]
No	93707 (99.8)	84011 (99.8)	9696 (99.8)		35615 (99.8)	31530 (99.8)	4085 (99.9)	
Yes	173 (0.18)	158 (0.19)	15 (0.15)		59 (0.17)	54 (0.17)	5 (0.12)	
**Abnormal liver or kidney function (%)**				<0.001[a]				<0.001[a]

No	89250 (95.1)	80166 (95.2)	9084 (93.5)		33807 (94.8)	29979 (94.9)	3828 (93.6)	
Yes	4630 (4.93)	4003 (4.76)	627 (6.46)		1867 (5.23)	1605 (5.08)	262 (6.41)	
**Gestational hyperuricemia (%)**				0.193[a]				<0.001[a]
No	93531 (99.6)	83864 (99.6)	9667 (99.5)		35415 (99.3)	31374 (99.3)	4041 (98.8)	
Yes	349 (0.37)	305 (0.36)	44 (0.45)		259 (0.73)	210 (0.66)	49 (1.20)	
**Gestational dyslipidemia (%)**				<0.001[a]				0.002[a]
No	67197 (71.6)	60611 (72.0)	6586 (67.8)		26559 (74.4)	23596 (74.7)	2963 (72.4)	
Yes	26683 (28.4)	23558 (28.0)	3125 (32.2)		9115 (25.6)	7988 (25.3)	1127 (27.6)	
**Elevated bile acids in pregnancy (%)**				<0.001[a]				<0.001[a]
No	92522 (98.6)	83137 (98.8)	9385 (96.6)		35369 (99.1)	31368 (99.3)	4001 (97.8)	
Yes	1358 (1.45)	1032 (1.23)	326 (3.36)		305 (0.85)	216 (0.68)	89 (2.18)	
**Ovarian cyst (%)**				0.603[a]				0.338[a]
No	91017 (97.0)	81611 (97.0)	9406 (96.9)		35119 (98.4)	31085 (98.4)	4034 (98.6)	
Yes	2863 (3.05)	2558 (3.04)	305 (3.14)		555 (1.56)	499 (1.58)	56 (1.37)	
**Uterine myoma (%)**				0.008[a]				0.620[a]
No	86656 (92.3)	77759 (92.4)	8897 (91.6)		32746 (91.8)	28983 (91.8)	3763 (92.0)	
Yes	7224 (7.69)	6410 (7.62)	814 (8.38)		2928 (8.21)	2601 (8.24)	327 (8.00)	
**Thyroid disorder (%)**				0.071[a]				0.527[a]
No	86192 (91.8)	77323 (91.9)	8869 (91.3)		31019 (87.0)	27476 (87.0)	3543 (86.6)	
Yes	7688 (8.19)	6846 (8.13)	842 (8.67)		4655 (13.0)	4108 (13.0)	547 (13.4)	
**HDP (%)**				<0.001[a]				<0.001[a]

No	87555 (93.3)	79313 (94.2)	8242 (84.9)		31841 (89.3)	28618 (90.6)	3223 (78.8)	
Yes	6325 (6.74)	4856 (5.77)	1469 (15.1)		3833 (10.7)	2966 (9.39)	867 (21.2)	
**DM/GDM (%)**				<0.001[a]				0.003[a]
No	80688 (85.9)	72650 (86.3)	8038 (82.8)		27654 (77.5)	24558 (77.8)	3096 (75.7)	
Yes	13192 (14.1)	11519 (13.7)	1673 (17.2)		8020 (22.5)	7026 (22.2)	994 (24.3)	
**Polyhydramnios (%)**				0.004[a]				0.522[a]
No	93241 (99.3)	83619 (99.3)	9622 (99.1)		35295 (98.9)	31244 (98.9)	4051 (99.0)	
Yes	639 (0.68)	550 (0.65)	89 (0.92)		379 (1.06)	340 (1.08)	39 (0.95)	
**Oligohydramnios (%)**				<0.001[a]				<0.001[a]
No	91887 (97.9)	82504 (98.0)	9383 (96.6)		34817 (97.6)	30902 (97.8)	3915 (95.7)	
Yes	1993 (2.12)	1665 (1.98)	328 (3.38)		857 (2.40)	682 (2.16)	175 (4.28)	
**Placental disorders (%)**				<0.001[a]				<0.001[a]
No	91040 (97.0)	82100 (97.5)	8940 (92.1)		33740 (94.6)	30090 (95.3)	3650 (89.2)	
Yes	2840 (3.03)	2069 (2.46)	771 (7.94)		1934 (5.42)	1494 (4.73)	440 (10.8)	

a Chi-square test;

BMI: Body mass index; ART: Assisted reproductive technology; GBS: Group B Streptococcus; HDP: Hypertensive disorders of pregnancy; DM: Diabetes mellitus; GDM: Gestational diabetes mellitus.
